# Differential Effect of Vaccine Effectiveness and Safety on COVID-19 Vaccine Acceptance across Socioeconomic Groups in an International Sample

**DOI:** 10.3390/vaccines9091010

**Published:** 2021-09-11

**Authors:** Stefania Kerekes, Mengdi Ji, Shu-Fang Shih, Hao-Yuan Chang, Harapan Harapan, Yogambigai Rajamoorthy, Awnish Singh, Shailja Kanwar, Abram L. Wagner

**Affiliations:** 1 Department of Epidemiology, School of Public Health, University of Michigan, 1415 Washington Heights, Ann Arbor, MI 48109, USA; stefaniakerekes@gmail.com (S.K.), mengdiji@umich.edu (M.J.); 2 Faculty of European Studies, Babeș-Bolyai University of Cluj-Napoca, 400090 Cluj-Napoca, Romania; 3 Department of Health Administration, College of Health Professions, Virginia Commonwealth University, Richmond, VA 23298, USA; shihs2@vcu.edu; 4 School of Nursing, College of Medicine, National Taiwan University, No. 1 Jen Ai Rd., Section 1, Taipei 100233, Taiwan; changh@ntu.edu.tw; 5 Department of Nursing, National Taiwan University Hospital, No. 7, Chung Shan S. Rd., Taipei 100225, Taiwan; 6 Medical Research Unit, School of Medicine, Universitas Syiah Kuala, Banda Aceh 23111, Indonesia; harapan@unsyiah.ac.id; 7 Tropical Disease Center, School of Medicine, Universitas Syiah Kuala, Banda Aceh 23111, Indonesia; 8 Department of Microbiology, School of Medicine, Universitas Syiah Kuala, Banda Aceh 23111, Indonesia; 9 Department of Economics, Faculty of Accountancy and Management, Universiti Tunku Abdul Rahman, Sungai Long Campus, Jalan Sungai Long, Cheras, Kajang 43000, Malaysia; yogambigai@utar.edu.my; 10 National Technical Advisory Group on Immunization Secretariat, National Institute of Health and Family Welfare, New Delhi, Delhi 110067, India; singhak.24@gmail.com; 11 Sapiens Public Health Solutions, New Delhi, Delhi 110092, India; drshailjakanwar@sphs.in

**Keywords:** vaccines, COVID-19 vaccines, cross-sectional studies, SARS-CoV-2, surveys and questionnaires, international sample, immunization

## Abstract

Controlling the spread of SARS-CoV-2 will require high vaccination coverage, but acceptance of the vaccine could be impacted by perceptions of vaccine safety and effectiveness. The aim of this study was to characterize how vaccine safety and effectiveness impact acceptance of a vaccine, and whether this impact varied over time or across socioeconomic and demographic groups. Repeated cross-sectional surveys of an opt-in internet sample were conducted in 2020 in the US, mainland China, Taiwan, Malaysia, Indonesia, and India. Individuals were randomized into receiving information about a hypothetical COVID-19 vaccine with different safety and effectiveness profiles (risk of fever 5% vs. 20% and vaccine effectiveness 50% vs. 95%). We examined the effect of the vaccine profile on vaccine acceptance in a logistic regression model, and included interaction terms between vaccine profile and socioeconomic/demographic variables to examine the differences in sensitivity to the vaccine profile. In total, 12,915 participants were enrolled in the six-country study, including the US (4054), China (2797), Taiwan (1278), Malaysia (1497), Indonesia (1527), and India (1762). Across time and countries, respondents had stronger preferences for a safer and more effective vaccine. For example, in the US in November 2020, acceptance was 3.10 times higher for a 95% effective vaccine with a 5% risk of fever, vs a vaccine 50% effective, with a 20% risk of fever (95% CI: 2.07, 4.63). Across all countries, there was an increase in the effect of the vaccine profile over time (*p* < 0.0001), with stronger preferences for a more effective and safer vaccine in November 2020 compared to August 2020. Sensitivity to the vaccine profile was also stronger in August compared to November 2020, in younger age groups, among those with lower income; and in those that are vaccine hesitant. Uptake of COVID-19 vaccines could vary in a country based upon effectiveness and availability. Effective communication tools will need to be developed for certain sensitive groups, including young adults, those with lower income, and those more vaccine hesitant.

## 1. Introduction

The WHO (World Health Organization) declared SARS-CoV-2 to be a pandemic on 11 March 2020 [1]. Throughout 2020, the outbreak spread quickly; for instance, between 1 August 2020 and 30 November 2020, the number of total cases worldwide increased from 17,396,943 to 62,391,667 [2].

Although the outbreak has had a differential impact across countries [3], controlling the spread of SARS-CoV-2 through vaccination is a goal of many countries. Fighting against anti-vaccine movements and misinformation will be a key part of promoting COVID-19 vaccinations globally, after insuring adequate access to vaccine supply [4]. The dynamics of vaccine hesitancy need to be understood globally because strong vaccine hesitancy could undermine the efforts to control the pandemic [4], and pockets of vaccine hesitancy and consequently lower vaccination coverage could reduce our ability to control the pandemic. The inclusion of study populations from different countries could help us understand the range of vaccine hesitancy and how a number of factors that vary geographically contribute to it [5]. Recognizing why some subgroups of population are more likely to be vaccine hesitant, will help in the development of immunization strategies to ensure adequate population coverage [5]. High vaccination coverage in the population could offer protection to unimmunized people through “herd immunity” [5].

Since the pandemic outbreak of SARS-CoV-2, many efforts have been made by governments, medical personnel and other institutions/organizations for stopping the spread of infections and limiting the burden of disease [6]. As SARS-CoV-2 spread rapidly through the world in 2020 [7], there was increased investment in research in COVID-19 vaccine development. As of May 14, 2021, there were 119 vaccine candidates, of which 15 have been granted emergency use authorization or approval [8]. Many of these vaccines have high efficacy [9]. For example, Novavax demonstrated 89% efficacy, Moderna showed 80% efficacy two weeks after the first dose and the Pfizer vaccine was among the best with 50% efficacy after the first dose [9]. Conversely, some vaccines have even lower efficacy, like CureVac with <50% efficacy [10]. mRNA vaccines like Pfizer and Moderna, may also be less effective against newer variants of SARS-CoV-2, like the delta variant [11]. Given supply or intellectual property constraints with mRNA vaccines like the Moderna or Pfizer vaccine, which have some of the most ideal effectiveness profiles of any COVID-19 vaccine, other vaccines with reduced effectiveness may be made available in locations, but the population may be accordingly less accepting of the vaccine [12]. The underlying theory of this hypothesis is the Health Belief Model, in which health behaviors like vaccination can be affected by perceived benefits (e.g., effectiveness) and barriers (e.g., safety) [13].

Overall, the acceptance for these vaccines depends not only on their widespread availability and convenience of access but also people’s confidence in vaccination [14]. Vaccine hesitancy was identified as a top global health threat by the WHO in 2019 [15]. Vaccine hesitancy can be dependent on various factors, including perceived safety and efficacy of the vaccine [16]. Vaccine acceptance can also vary by socioeconomic factors like age, gender, and income [17,18,19].

There may be worldwide variations in COVID-19 vaccine acceptance, particularly as roll-out of the vaccine is uneven across the globe. In a survey conducted in China, scientists observed that vaccine acceptancy was very high, especially among health care workers and the main reason was the high trust in the central government [20,21]. Among Americans, the general acceptance rate was highly influenced by several factors like race, political affiliation, news on media, social status, and others [22,23]. The highest acceptance rate was observed among the elderly and those with high income and high education [22,23]. A common motive of vaccine hesitancy was the fear of the side effects [22]. In Indonesia, health care workers showed a higher rate of vaccine acceptancy than the general population without medical expertise [24]. In Malaysia and India, previous surveys demonstrated that the vaccine acceptance rate was also high [18,24,25].

The differential availability across countries of different COVID-19 vaccine types, which have varying safety and effectiveness profiles, may make it difficult to quickly attain a high level of vaccine uptake, even when the vaccine is available. The aim of this study was to characterize how vaccine safety and effectiveness impacts acceptance of a vaccine, and whether this impact varied over time or across socioeconomic groups. We hypothesize that individuals will prefer safer and more effective vaccines, but that this degree of preference could vary significantly across countries. This information contributes to our understanding of COVID-19 vaccine decision-making on a global scale and can identify potential pitfalls in the roll-out of COVID-19 vaccines with lower effectiveness or safety.

## 2. Materials and Methods

### 2.1. Study Population

This study is part of a larger project looking at resiliency and adherence to public health countermeasures during the COVID-19 pandemic. We conducted several waves of cross-sectional surveys (i.e., different samples each wave) between March and November 2020 (Table 1), with six countries having surveys conducted both in August and November 2020, in six countries/regions: the US, China, Taiwan, Malaysia, Indonesia, and India. Using random assignment, individuals received information about hypothetical COVID-19 vaccine with different safety and effectiveness profiles (risk of fever 5% vs. 20% and vaccine effectiveness 50% vs. 95%). We chose the August – November time frame because all countries were represented during this period. We selected a sample of individuals through cross-sectional surveys of panelists curated by the market survey research firm Dynata. These are opt-in samples, with panels formed from individuals selected through social media and advertisements. To be part of the sample, individuals had to be ≥18 years or older in all places except Taiwan, where they had to be ≥20 years. We eliminated individuals who took shorter than 180 s on the survey, which we judged to be the minimal adequate time to thoughtfully complete the survey. We also only included individuals who completed most of the survey (up to the start of the demographic questions at the end). We also excluded individuals who identified as a gender other than male or female (N = 29). In order to obtain a wide distribution of individuals, we set up age and gender quotas roughly proportional to their size within the larger population. Subsequently, we raked weights for each individual based on their age, gender, and region of country. All data are available online at: https://doi.org/10.3886/E130422V2 (accessed on 15 June 2021). Questionnaires, and details of the sampling scheme, are available at: https://doi.org/10.6084/m9.figshare.14792058.v2 (accessed on 17 June 2021).

We attempted to obtain a sample size of 800 for each country for each wave of data collection. With an alpha of 0.05 and a power of 80%, and a proportion of 50% (a statistically conservative estimate of what proportion of the population supports vaccination), the margin of error will be 4%. This margin of error would allow us to assess substantial trends over time. 

### 2.2. Data Collection

Respondents received this question: “A vaccine is currently not available for the new coronavirus strain (called SARS-CoV-2 and which causes COVID-19). Imagine that a new coronavirus vaccine has just been developed. It has received the same testing as the adult influenza vaccine. The government is offering it as a free and optional vaccine. Would you accept a coronavirus vaccine, which is (95%) effective, with a (5%) chance of a side effect like fever? (95%) effective means that there is a (95%) reduction in disease among those vaccinated compared to those unvaccinated.” Participants were randomized to one of four groups, with effectiveness varying between 95% and 50%, and risk of side effect varying between 5% and 20%. These experimental groups are the main independent variable, and acceptance of a COVID-19 vaccine is the main outcome.

### 2.3. General Adult Vaccine Hesitancy

We measured general vaccine hesitancy on a 10-item scale, whose properties were previously evaluated in Chinese and US studies [26]. Briefly, each item was on a 5-point scale. We summed the items (with a possible range of 10 to 50), and designated individuals as hesitant if they had a score of 25 or higher. Vaccine hesitancy was added as an independent variable into the multivariable regression models.

### 2.4. Socioeconomic and Demographic Factors

We explored vaccine acceptance across three socioeconomic and demographic factors: age, gender, and income. For age, we split the sample into three groups: 18–34, 35–54, and 55 and above. For gender, individuals could self-report male, female, or other, with others being excluded from the analysis. For monthly household income, individuals responded to several categories. We categorized these into “higher” and “lower” income based roughly on a cut-off of $2000 per month, based on a purchasing power parity currency conversion. The lower category was ≤$2000 in the US, ≤7500 CNY in China, ≤30,000 TWD in Taiwan, ≤40,000 INR in India, and ≤3000 MYR in Malaysia.

### 2.5. Statistical Analysis

Our initial analysis was a test of the experimental effect of vaccine profile attributes on vaccine acceptance. In our first set of models, we constructed separate logistic regression models for each country and for each wave of data collection. The second set of models tested changes between August and November. For this, we combined data from all countries and waves, and we placed an interaction term between an indicator variable for wave and independent variables, including the vaccine profile attributes. In the third set of models, we examined differences in the effect of vaccine profile attributes by specifying an interaction term between sociodemographic variables and the vaccine profile attributes. In this third set of models, we report marginal estimates (i.e., least square means), which are predicted margins on the logit scale, balanced over the other covariates in the model. The p-value for the interaction term between the experimental vaccine profile and other variables thus indicates whether the effect of the experimental vaccine profile differed by group.

The first set of models only included the experimental conditions (the vaccine profile attributes) in the model. The second model also adjusted for age, gender, income, and vaccine hesitancy. All models used weights so that the survey respondents matched the general population’s distribution by age, gender, and region of country. In the US, model weights were also based on race/ethnicity. Details about the weight construction, including sources of population information, are available at: https://doi.org/10.3886/E130422V2 (accessed on 15 June 2021). The models output odds ratios (OR) and 95% confidence intervals (CI). We used survey procedures that accounted for clustering between countries, and incorporated weights. We assessed significance at an alpha = 0.05 level, and used SAS version 9.4 (SAS Institute, Cary, NC, USA).

## 3. Results

In total, 12,886 participants were enrolled in the studies from six countries in five waves from March, June, Aug, October, and November in 2020, including the United States (4050), China (2797), Indonesia (1507), India (1762), Malaysia (1492), and Taiwan (1278). Participants were divided into lower and higher income levels based on the $2000 monthly salary by PPP currency conversion. Most of the respondents from the U.S. (77.2%), China (89.5%), Malaysia (71.4%), and Taiwan (89.1%) were from the higher income level. More than half of the participants from Indonesia (61.7%) and India (53.6%) were from the lower-income group.

Preferences of the COVID-19 vaccine with different effectiveness and safety in different waves were shown in Table 2. Respondents from both waves from all countries had stronger preferences for a safer, more effective vaccine, but, in general, there were fewer differences by safety than by effectiveness. For example, in the US in Nov 2020, changing a 50% effective vaccine from having 20% risk of fever to 5% risk of fever did not significantly change acceptance (OR: 1.12, 95% CI: 0.76, 1.64), whereas a change from 50% to 95% effectiveness did lead to more acceptance (OR: 1.95, 95% CI: 1.28, 2.97).

Changes in vaccine acceptance over time are shown in Table 3. Across all countries, there was an increase in the effect of the vaccine profile over time (*p* < 0.0001), such that were reduced preferences for the least effective and safe vaccine in November 2020 compared to August 2020. Patterns of acceptance by age, gender, and income did not vary between August and November 2020. 

Overall, vaccine acceptance increases from 75% in the 50% effective vaccine with a 20% risk of fever, to 88% in the 95% effective vaccine with a 5% risk of fever (Table 4). The impact of the vaccine profile on acceptance, i.e., the difference in acceptance on the odds ratio scale, differed by age, income, and vaccine hesitancy. Older individuals were more sensitive to a worse profile compared to younger individuals (for example, 85%–88% would accept the most ideal vaccine, regardless of age, but only 67% of those ≥55 years would accept a 50% effective vaccine with a 5% risk of fever, vs 80% of those 18–34 years). By income, there was similar acceptance of less ideal vaccines, but greater acceptance of the most ideal vaccine in higher income groups. There was also greater acceptance of vaccines in the group not vaccine hesitant.

## 4. Discussion

This study has investigated how vaccine safety and effectiveness influences vaccine acceptancy among different socioeconomic groups over time. Individuals’ preferences for a more effective and a safer vaccine are obvious and well documented previously [27,28], but if there is a large difference between acceptance across different levels of safety or effectiveness, it could mean diminished uptake of the vaccine if these attributes are believed to be low. From our findings, the likelihood of getting the vaccine changed according to income, across age, and over time. Demographic differences in uptake could also point to clustering of non-vaccination, which could further diminish the effectiveness of COVID-19 vaccination programs [29].

Other studies have pointed out stubborn rates of vaccine refusal despite the availability of COVID-19 vaccines [30,31]. There could be a number of reasons for this. A study showed that (perceived) “effectiveness” is the most important characteristic for a vaccine to be accepted by the population, and politicized approval of vaccines is connected with more hesitancy in vaccine uptake. Their results also showed that people would choose the most effective vaccine with least side effects [32]. In the absence of such a choice, it could be that the lack of a more effective vaccine could lead to lower vaccination coverage within a community. This could arise, for example, if low- and middle-income countries are offered or develop vaccines less effective than COVID-19 vaccines currently available in high income countries.

We found that socioeconomic status could moderate vaccine decision-making. For example, individuals with a lower income were less accepting of a COVID-19 vaccination, but this was most readily apparent with the safest and most effective vaccine (e.g., the higher income was 89% accepting of the safest and most effective vaccine (95% CI: 82%, 94%), and the lower income group was 83% accepting (95% CI: 72%, 90%). This could be due to lower health literacy in lower income groups. Studies examining education have found mixed relationships between educational attainment and vaccination status. For example, in one study there was no specific association between economic hardship, education and vaccine hesitancy [32], but other researchers have not found a clear connection between high-education and vaccine hesitancy [33,34]. In studies of pediatric vaccination, lower education in both mother and father is also a strong predictor of vaccine refusal for their children [34]. Education might affect the decision of vaccine uptake in a way that people with higher-education background might use selected sources of information, and preventing more or certain types of information about vaccine safety could increase acceptance [31]. Consumption of this type of information could vary by socioeconomic or demographic group.

Gender and age played an important role in vaccine hesitancy. According to one study, men are less hesitant in receiving a vaccine then women and older people are more willing to receive the vaccine [33]. In contrast, our study showed no substantial differences by gender, or by gender over time. In terms of age, we found that younger adults were more likely to accept a vaccine, and that this difference did not vary across wave. In several other studies there was an inverse relationship between age and willingness of getting vaccinated. For example, studies have shown women and elderly residents were less likely to accept a COVID-19 vaccine, while men and younger people were equally receptive for getting a shot [30,32]. In other studies, older people were more likely to accept the vaccine than younger people [18,34]. Variations in vaccine acceptance by age could come from differences in educational status or in perceived risk across generations. Overall, these studies point to the need to better understand the local circumstances of vaccination acceptance, as these sociodemographic differences may vary across countries.

### Limitations

There are several limitations to this study. We use opt-in samples, and so the study population is biased to a more affluent, internet-accessing population. This study did not contain probability samples, which are very difficult to obtain in many low- and middle-income countries. Therefore, the results should not be considered representative of any one country and should be confirmed in additional studies with more robust samples. Additionally, we did not ascertain certain important variables, including educational status, marital status, size of the family unit, or numbers and age of children. It is possible that social desirability bias could have affected some responses. We measured vaccine hesitancy dichotomously, but it is a complex phenomenon that may not be fully captured with our variable. We also note that our study’s time frame (August–November 2020 for all countries) occurred during rapidly changing epidemiological circumstances and vaccine development. Opinion about a vaccine could have changed across this time. In contrast to a longitudinal study, we were not able to look at time-varying changes within an individual. The strength of our study was a robust experimental design and the inclusion of various countries.

## 5. Conclusions

Our study provides additional support for understanding vaccine acceptancy worldwide. When examining acceptance of COVID-19 vaccination by general vaccine hesitancy, we found that those who were more vaccine hesitant were more sensitive to the vaccine safety and effectiveness profile. This means that increased general concerns about vaccines, a trend that seems to be intensifying in recent years, could mean that the population is more particular about what vaccine they want to receive [30]. However, in a situation where a less effective vaccine is only available, it could mean continued propagation of an outbreak as vaccination efforts stall.

## Figures and Tables

**Table 1:** Demographic characteristics of study participants.

	USA	China	Taiwan	Malaysia	Indonesia	India
Overall	N = 4050	N = 2797	N = 1278	N = 1492	N = 1507	N = 1762
**Wave**						
Mar 2020	691 (20.0%)	1070 (33.3%)	--	--	--	--
Jun 2020	655 (19.9%)	--	--	--	--	--
Aug 2020	782 (20.0%)	788 (33.3%)	645 (50.0%)	757 (50.0%)	716 (49.8%)	805 (50.0%)
Oct 2020	936 (20.0%)	--	--	--	--	--
Nov 2020	986 (20.0%)	939 (33.3%)	633 (50.0%)	735 (50.0%)	791 (50.2%)	957 (50.0%)
**Age (years)**						
18–34	1057 (33.8%)	963 (34.1%)	523 (28.8%)	710 (42.8%)	702 (42.1%)	795 (43.2%)
35–54	1400 (35.0%)	1165 (39.2%)	607 (43.2%)	670 (40.1%)	676 (40.5%)	666 (38.1%)
≥55	1593 (31.2%)	669 (26.7%)	148 (28.0%)	112 (17.1%)	129 (17.4%)	301 (18.8%)
**Gender**						
Female	2128 (50.6%)	1386 (48.8%)	701 (52.1%)	713 (48.3%)	713 (49.1%)	824 (48.2%)
Male	1922 (49.4%)	1411 (51.2%)	577 (47.9%)	779 (51.7%)	749 (50.1%)	938 (51.8%)
**Income**						
<$2000 equivalent	891 (22.8%)	276 (10.5%)	114 (11.0%)	391 (28.6%)	908 (61.7%)	922 (53.6%)
≥$2000 equivalent	3155 (77.2%)	2489 (89.5%)	1113 (89.1%)	1100 (71.4%)	599 (38.3%)	840 (46.5%)
**Vaccine hesitant**						
No	2371 (57.4%)	1985 (72.9%)	395 (40.0%)	904 (59.3%)	863 (57.0%)	1200 (68.3%)
Yes	1626 (42.6%)	758 (27.1%)	593 (60.0%)	588 (40.7%)	644 (43.0%)	562 (31.7%)

**Table 2:** Effect of vaccine effectiveness (VE) and risk of fever on acceptance of a COVID-19 vaccine using logistic regression models that output odds ratios and 95% confidence intervals, in six countries, August–November, 2020.

	50% VE, 20% Fever Risk	50% VE, 5% Fever Risk	95% VE, 20% Fever Risk	95% VE, 5% Fever Risk
USA, Mar 2020	ref	1.59 (0.94, 2.67)	2.67 (1.37, 5.20)	3.81 (1.97, 7.36)
USA, Jun 2020	ref	0.97 (0.60, 1.57)	1.88 (1.06, 3.31)	2.33 (1.35, 4.02)
USA, Aug 2020	ref	1.22 (0.77, 1.94)	1.70 (1.00, 2.90)	1.64 (0.98, 2.73)
USA, Oct 2020	ref	0.86 (0.58, 1.29)	1.95 (1.28, 2.97)	3.64 (2.36, 5.63)
USA, Nov 2020	ref	1.12 (0.76, 1.64)	2.53 (1.71, 3.74)	3.10 (2.07, 4.63)
China, Mar 2020	ref	0.82 (0.38, 1.78)	0.86 (0.39, 1.90)	0.93 (0.41, 2.11)
China, Aug 2020	ref	1.03 (0.53, 2.01)	2.51 (1.17, 5.37)	3.23 (1.44, 7.23)
China, Nov 2020	ref	0.84 (0.49, 1.46)	2.00 (1.04, 3.84)	2.93 (1.44, 5.98)
Taiwan, Aug 2020	ref	0.89 (0.49, 1.63)	3.08 (1.51, 6.25)	2.13 (1.10, 4.11)
Taiwan, Nov 2020	ref	1.08 (0.63, 1.85)	3.76 (2.11, 6.73)	4.40 (2.41, 8.02)
Malaysia, Aug 2020	ref	1.27 (0.74, 2.18)	2.76 (1.49, 5.10)	2.81 (1.48, 5.34)
Malaysia, Nov 2020	ref	1.31 (0.74, 2.31)	3.48 (1.97, 6.16)	4.53 (2.38, 8.62)
Indonesia, Aug 2020	ref	1.11 (0.55, 2.24)	4.82 (2.31, 10.06)	2.13 (0.97, 4.64)
Indonesia, Nov 2020	ref	0.85 (0.49, 1.48)	2.21 (1.05, 4.68)	1.05 (0.56, 1.97)
India, Aug 2020	ref	0.97 (0.45, 2.07)	3.40 (1.34, 8.64)	3.29 (1.26, 8.61)
India, Nov 2020	ref	0.96 (0.53, 1.76)	3.15 (1.57, 6.32)	2.31 (1.32, 4.02)

**Table 3:** Change in the effect of vaccine profile on vaccine acceptance between August and November, 2020, in six countries.

	August Wave OR (95% CI)	November Wave OR (95% CI)	*p*-Value of Interaction ^a^
**Vaccine profile**			<0.0001
50% VE, 20% fever risk	ref	ref	
50% VE, 5% fever risk	1.09 (0.99, 1.21)	1.00 (0.91, 1.10)	
95% VE, 20% fever risk	2.88 (1.97, 4.21)	2.61 (1.97, 3.45)	
95% VE, 5% fever risk	2.39 (1.76, 3.23)	2.62 (1.66, 4.13)	
**Vaccine hesitant**			0.5575
No	ref	ref	
Yes	0.26 (0.19, 0.36)	0.25 (0.18, 0.34)	
**Age (years)**			0.627
18–34	1.46 (1.02, 2.08)	1.35 (0.85, 2.14)	
35–54	1.28 (1.07, 1.54)	1.10 (0.71, 1.69)	
≥55	ref	ref	
**Gender**			0.9546
Male	ref	ref	
Female	0.93 (0.64, 1.37)	0.93 (0.70, 1.25)	
**Income**			0.7793
<$2000 equivalent	ref	ref	
≥$2000 equivalent	1.06 (0.74, 1.51)	1.12 (0.66, 1.88)	

Notes: VE, vaccine effectiveness. ^a^ Table portrays logistic regression model with an interaction term between each variable and wave (August vs November). Columns represent the model with the reference group for the wave interaction changed. *p*-value is from the interaction between variable and wave, testing change in strength of association between August and November wave. Significant results mean that there is a significant difference in the strength of the OR between August and November.

**Table 4:** Marginal estimates of vaccine acceptance (and 95% confidence intervals) across different vaccine profiles as predicted by logistic regression models, stratified by demographic characteristics in six countries.

	50% VE, 20% Fever Risk	50% VE, 5% Fever Risk	95% VE, 20% Fever Risk	95% VE, 5% Fever Risk	*p*-Value ^a^
**Overall**	75% (63%, 80%)	76% (66%, 83%)	89% (81%, 94%)	88% (81%, 92%)	
**By wave**					<0.0001
Aug 2020	77% (68%, 84%)	78% (68%, 85%)	89% (82%, 94%)	88% (80%, 93%)	
Nov 2020	71% (56%, 82%)	71% (59%, 81%)	86% (78%, 92%)	87% (80%, 91%)	
**By age**					<0.0001
18–34	76% (68%, 82%)	80% (76%, 84%)	87% (76%, 93%)	88% (81%, 93%)	
35–54	73% (62%, 82%)	74% (59%, 85%)	88% (80%, 93%)	87% (80%, 92%)	
≥55	72% (51%, 86%)	67% (54%, 77%)	90% (84%, 94%)	85% (78%, 90%)	
**By gender**					0.1864
Male	73% (60%, 83%)	75% (60%, 85%)	87% (76%, 94%)	85% (74%, 92%)	
Female	75% (64%, 84%)	75% (67%, 80%)	88% (83%, 92%)	89% (85%, 92%)	
**By income**					<0.0001
<$2000 equivalent	75% (64%, 83%)	75% (64%, 84%)	86% (75%, 93%)	83% (72%, 90%)	
≥$2000 equivalent	74% (60%, 84%)	74% (63%, 83%)	89% (81%, 94%)	89% (82%, 94%)	
**By vaccine hesitancy**					<0.0001
No	84% (77%, 89%)	85% (76%, 90%)	95% (92%, 97%)	96% (92%, 98%)	
Yes	61% (47%, 73%)	64% (53%, 74%)	78% (66%, 87%)	75% (64%, 84%)	

Notes: VE, vaccine effectiveness. ^a^ Interaction between vaccine profile and demographic variable.

## Data Availability

All data are available at OpenICPSR: All data are available online at: https://doi.org/10.3886/E130422V2, accessed on 10 September 2021. Code associated with this paper is available at: 10.6084/m9.figshare.16547877, accessed on 10 September 2021.
